# Using Combinations of Both Clinical and Radiographic Parameters to Develop a Diagnostic Prediction Model Demonstrated an Excellent Performance in Early Detection of Patients with Blount’s Disease

**DOI:** 10.3390/children8100890

**Published:** 2021-10-06

**Authors:** Nath Adulkasem, Jidapa Wongcharoenwatana, Thanase Ariyawatkul, Chatupon Chotigavanichaya, Kamolporn Kaewpornsawan, Perajit Eamsobhana

**Affiliations:** Department of Orthopaedic Surgery, Faculty of Medicine, Siriraj Hospital, Mahidol University, Bangkok 10700, Thailand; adulkasem.n@gmail.com (N.A.); jidapa.wongcha@gmail.com (J.W.); thanaseortho@gmail.com (T.A.); chatuponc@gmail.com (C.C.); kamolporn.kaewpornsawan@gmail.com (K.K.)

**Keywords:** genu varum, infantile Blount’s disease, physiologic bowlegs, prediction, diagnosis

## Abstract

Early identification of pathological causes for pediatric genu varum (bowlegs) is crucial for preventing a progressive, irreversible knee deformity of the child. This study aims to develop and validate a diagnostic clinical prediction algorithm for assisting physicians in distinguishing an early stage of Blount’s disease from the physiologic bowlegs to provide an early treatment that could prevent the progressive, irreversible deformity. The diagnostic prediction model for differentiating an early stage of Blount’s disease from the physiologic bowlegs was developed under a retrospective case-control study from 2000 to 2017. Stepwise backward elimination of multivariable logistic regression modeling was used to derive a diagnostic model. A total of 158 limbs from 79 patients were included. Of those, 84 limbs (53.2%) were diagnosed as Blount’s disease. The final model that included age, BMI, MDA, and MMB showed excellent performance (area under the receiver operating characteristic (AuROC) curve: 0.85, 95% confidence interval 0.79 to 0.91) with good calibration. The proposed diagnostic prediction model for discriminating an early stage of Blount’s disease from physiologic bowlegs showed high discriminative ability with minimal optimism.

## 1. Introduction

Pediatric genu varum deformity, also known as bowlegs, is one of the most frequent causes of parental concerns in children aged one to three years old [1]. Although the vast majority of cases are physiological conditions, which will spontaneously resolve with growth, pathological causes of genu varum deformity, such as Blount’s disease, should be distinguished [1,2]. In contrast to the physiologic bowlegs, Blount’s disease is a progressive condition causing an irreversible severe varus deformity of the knee if the treatment initiation is delayed [3]. Even though the diagnosis can be easily established upon radiographic changes of the medial proximal tibial physis described by Langenskiöld [3], an absence of substantial radiographic abnormalities in the early stage of the disease may cause problems in making an accurate early diagnosis. This is especially true for primary care physicians, who are often the first to encounter the patients and thus play a crucial role in the early identification of Blount’s disease [4,5].

To address this diagnostic challenge, several radiographic parameters have been proposed for differentiating Blount’s disease and physiologic bowlegs, such as the classic metaphyseal-diaphyseal angle (MDA) [6], the rate of MDA change [4], and the medial metaphyseal beak angle (MMB) [7]. Nevertheless, these radiographic parameters vary among different patient characteristics (e.g., age group and other risk factors), and therefore the accuracy of these diagnostic parameters has been questioned by several studies [4,8,9].

One strategy to improve the accuracy in making an early diagnosis is by creating a clinical prediction rule (CPR), a formal combination of several predictive factors using statistical modeling, which will predict the probability or likelihood of developing radiographic abnormalities in medial proximal tibial physis, specifically for each patient [10]. In clinical practice, the diagnostic prediction provided by the CPR might be beneficial in several circumstances. For example, the prediction could be used by primary care physicians or pediatricians to provide a prompt referral to pediatric orthopaedists in patients with high risk for Blount’s disease. In addition, an early treatment initiation could be justified by pediatric orthopaedists according to the patient’s individual risk. Accordingly, the aim of this study was to develop and validate a diagnostic clinical prediction model for distinguishing an early stage of Blount’s disease from the physiologic bowlegs, which could improve the diagnostic accuracy in an early stage of the disease.

## 2. Materials and Methods

### 2.1. Study Design

Development and internal validation of a diagnostic prediction model were conducted via a retrospective observational case-control study of children aged one to four years who presented with bowlegs at the outpatient pediatric orthopedic clinic of a tertiary university-affiliated hospital from January 2000 to December 2017. This study was conducted in accordance with the declaration of Helsinki [11] and has been approved by the hospital’s institutional review board (COA no. 594/2564).

### 2.2. Study Patients

Patients within the ages of one to four years initially presented with genu varum deformity who later diagnosed as infantile Blount’s disease with Langenskiöld stage II were included during the study period. We excluded patients whose medial proximal tibial physis radiographic abnormalities were already developed in an initial radiographic study. The objective of this study was to develop a diagnostic prediction tool to distinguish an early stage of Blount’s disease from physiologic bowlegs. Therefore, patients with other causes of pathological bowlegs, including metabolic bone disease, focal fibrocartilaginous dysplasia, and other orthopedic or medical lower extremities conditions—with or without previous treatment—were excluded from the study. A control series of physiologic bowlegs patients with the same age group were retrieved and allocated from the medical records. All included study patients had complete initial and follow-up radiographic studies of the lower extremities.

### 2.3. Study Variables and Candidate Predictors

The patient’s initial demographic and clinical information (patient’s ages, sex, affected sides, and body mass index (BMI)) were retrieved from our center’s electronic medical record system. Radiographic measurements were taken from each patient’s initial radiographic work-up. The femoro-tibial angle (FTA) [7], MDA [6], and MMB [7] were measured and recorded from an initial radiographic study. All measurements were performed twice by two independent investigators, both of whom were blinded to the clinical endpoint to prevent review bias.

### 2.4. Clinical Endpoints

The definitive diagnosis of Blount’s disease in this study was defined as the development of radiographic change in the medial proximal tibial physis as described by Langenskiöld after the patient’s initial presentation during the study period. According to Langenskiöld, Blount’s disease is definitely diagnosed after the identification of a progressive proximal tibia varus deformity with a medial proximal tibial physis osteochondrosis [3]. Therefore, in this study, two pediatric orthopaedists independently diagnosed Blount’s disease by comparing baseline radiographic studies with subsequent radiographic studies. In case of any disagreement between investigators, the diagnosis was discussed with and decided by a third senior investigator.

### 2.5. Statistical Methods

#### 2.5.1. Study Size Estimation

According to the standard recommendation, a minimum of 10 events of interest is required for each included predictor [12]. In this study, seven candidate predictors were preselected, and 70 patients diagnosed with Blount’s disease were required.

#### 2.5.2. Fundamental Statistical Analysis

All statistical analyses were performed using STATA (version 14.0; StataCorp, LLC, College Station, TX, USA). Data distribution patterns were identified using histogram and Shapiro-Wilk test. Normally distributed continuous variables are described as means ± standard deviation (SD), and they were compared using an independent *t*-test. Non-normally distributed variables are presented as medians and interquartile ranges (IQR) and were compared using the Mann-Whitney U test. Counts and percentages were used to describe categorical data, and these variables were compared using Fisher’s exact probability test. Statistical significance for all analyses was set at a *p*-value less than 0.05 and statistical power of 0.80.

#### 2.5.3. Model Development

The multivariable diagnostic prediction model in this study was developed and reported according to the Transparent Reporting of a multivariable prediction model for Individual Prognosis or Diagnosis (TRIPOD) statement [12].

- Missing data management

The multiple imputation (MI) technique was used to impute the missing variables to improve the accuracy and statistical power of the model [13]. Predictive mean matching (PMM) methods were performed using the complete recorded variable to impute the missing variable [13]. As a result, a total of 10 datasets were imputed to preserve the uncertainty and variability of the imputed dataset.

- Continuous predictors management

To fulfill the linearity assumption of the logistic regression analysis, all continuous predictors were categorized according to the findings of previous studies. Physiologic resolution of bowlegs regularly begins between the ages of 18 and 30 months [1]. For this reason, we categorized patient’s ages at the midpoint of this range (24 months). High BMI (greater than 23 kg/m^2^) was reported to be associated with Blount’s disease [14,15]. The normal FTA among children aged 2 to 4 years was reported to be 5° [16]. The MDA was categorized into <11°, 11° to 16°, and >16° [6]. The MMBs greater than 122° were identified as an independent predictor for Blount’s disease [7].

- Predictive model development

The predictive model was developed using a multivariable logistic regression analysis with pre-specified predictors including age, gender, BMI, FTA, MDA, and MMB. The stepwise backward elimination procedure was performed by evaluating the effect size, the level of significance, and the clinical relevance of each predictor to create a parsimonious predictive model.

- Model performance and internal validation

The discriminative ability of the final predictive model was assessed using the area under the receiver operating characteristic (AuROC) curve. According to the TRIPOD statement, the model calibration was reported using a calibration curve demonstrating the actual observed risk and the level of risk predicted by the model [12]. Internal validation using the bootstrap resampling method with 100 replications was performed to determine the level of model optimism.

- Model presentation

A predictive scoring system was derived from the final multivariable logistic regression model. The regression coefficient (β) of each item was transformed into a weighted score by rounding up the fraction of each coefficient to the lowest coefficient in the model. The total score was categorized into three recommendation levels (low, moderate, and high risk for Blount’s disease) to help guide physicians in decision-making. The positive likelihood ratio (LHR+) of the low-risk group should be <1, while the negative likelihood ratio (LHR−) should be >5 to accurately identify physiologic bowlegs patients. In contrast, the high-risk group LHR+ value in the high-risk group was set at >5, which indicates a greater chance of Blount’s disease diagnosis and the potential need for treatment. Patients with a borderline LHR+ value close to one were classified as the moderate-risk group, which is recommended for close observation and serial radiographic study.

## 3. Results

A total of 158 lower extremities from 79 children were included in the study. Of those, 28 (35.4%) had bilateral Blount’s disease, 28 (35.4%) had unilateral involvement (9 (11.4%) right side, and 19 (24.1%) left side), and 23 (29.1%) had bilateral physiologic bowlegs (Table 1). Demographic and clinical information on lower extremities categorized by the study endpoint (Blount’s disease (*n* = 84) and physiologic bowlegs (*n* = 74)) were summarized and compared. Patients diagnosed with Blount’s disease were significantly older (27 ± 5.2 vs. 24.9 ± 6.9 months, *p* = 0.030), and had greater FTA (13.5 ± 6.2° vs. 9.2 ± 7.3°, *p* < 0.001), greater MDA (14.5 ± 4.0° vs. 10.0 ± 4.4°, *p* < 0.001), and higher MMB (127.4 ± 6.1° vs. 118.3 ± 6.2, *p* < 0.001) (Table 2). The distribution of variables after categorization with a pre-specified cut-off point is presented. Of all observations, only patient BMI information was missing for 62 (39.2%) patients. Therefore, multiple imputation analysis was performed using all other predictors (age, gender, FTA, MDA, and MMB) as independent predictors by the PMM method. The interobserver reliability of radiographic parameter measurement showed a substantial agreement with an ICC greater than 0.9 for all radiographic measurements.

**Figure 1:**
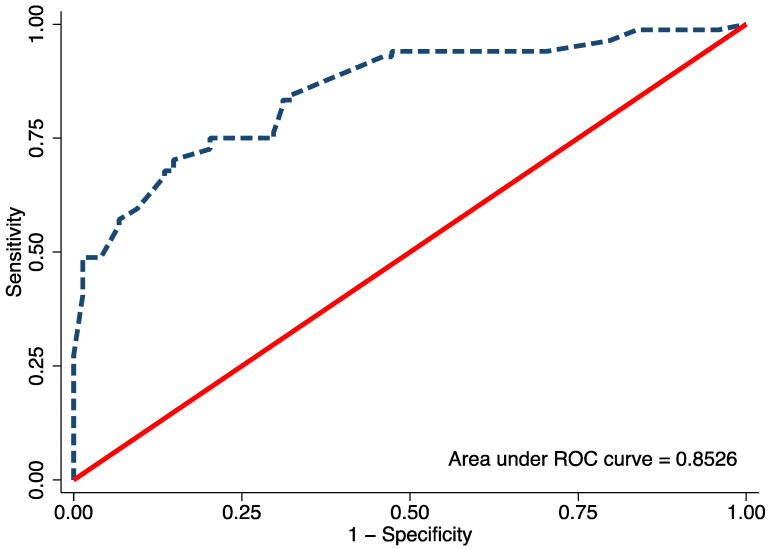
The area under the receiver operating characteristic (ROC) of the final proposed diagnostic model, including age, body mass index, metaphyseal-diaphyseal angle, and medial metaphyseal beak angle.

**Figure 2:**
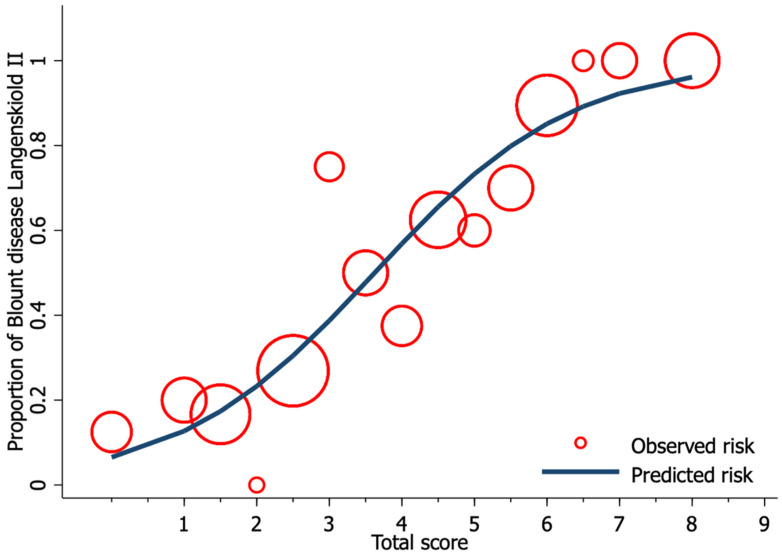
Calibration plot of the observed risk (red circle) and predicted risk (navy line) of Blount’s disease relative to total score from the proposed diagnostic model.

Univariable logistic regression analysis revealed age, FTA, MDA, and MMB to be statistically significant predictors of Blount’s disease (Table 3). Nevertheless, all candidate predictors were included in the full model multivariable logistic regression analysis using the multiple imputed datasets. Of the six predictors, three were identified as independent predictors including age ≥ 24 months (mOR 2.75, 95% CI 1.09 to 6.95, *p* = 0.03), MDA > 16° (mOR 11.65, 95% CI 2.44 to 55.63, *p* = 0.002), and MMB ≥ 122° (mOR 4.47, 95% CI 1.59 to 11.52, *p* = 0.005). However, previous studies identified BMI as a strong predictor for Blount’s disease. Therefore, after discussion with all investigators, we decided to include patient BMI along with other independent predictors in the final predictive model. The discriminative ability of the final model was found to be excellent, with an AuROC of 0.85 (95% CI 0.79 to 0.91) (Figure 1). The regression coefficient for each predictor from the final model was then transformed into a weighted score (Table 4). The scoring scheme with a total score from 0 to 8 was then classified into three risk groups for clinical implementation. The groups were defined as low-risk, moderate-risk, and high-risk based on a total score > 2.5, within 2.5 to 5.5, or >5.5, respectively (Table 5). The mean total score was significantly different between the Blount’s disease group and the physiologic bowlegs group (5.2 ± 0.2 vs. 2.5 ± 0.2, *p* < 0.001). Model calibration is presented via calibration plots, as recommended by the TRIPOD statement in Figure 2 [12]. Internal validation using the bootstrap resampling method revealed an optimism of 0.018 (range 0.018 to 0.028).

## 4. Discussion

This study identified patient clinical information (age and BMI) and lower extremity radiographic parameter abnormality (MDA and MMB) as independent predictors of Blount’s disease with Langenskiöld stage II. The developed scoring system that subcategorizes patients as low-, moderate-, or high-risk for Blount’s disease will assist clinicians with management decision-making when they encounter a pediatric patient presenting with genu varum.

Early diagnosis and management of Blount’s disease is recommended to prevent irreversible damage to the proximal medial tibial physis, which leads to either intra-articular or extra-articular deformities of the proximal tibia [7,17,18]. Current management for this condition focuses on lower extremity mechanical axis realignment to unload the proximal medial tibial physis using operative and nonoperative procedures [7,19]. However, this management protocol was found to be ineffective in patients with late presentation Blount’s disease due to the formation of physeal bony bridges (as described in Langenskiöld stages IV to VI) [3] that require more complicated procedures such as the bony bridge resection, joint elevation procedure, or gradual correction [20].

Several clinical risk factors for Blount’s disease have been reported. An atypical age of initial patient presentation should heighten suspicion for pathological causes of the bowlegs [1,16,21]. Consequently, older aged patients (>2 years) who present with bowlegs have greater odds of being diagnosed with Blount’s disease, as presented in this study. The proximal medial physis pathology observed in Blount’s disease could result from the repetitive stress on the physis in overweight patients with a higher BMI [22,23,24]. Therefore, we made the decision to include BMI in the final model analysis (even though we did not find it to be statistically significant) to improve the clinical relevance of the analysis.

MDA is a well-known radiographic predictor of Blount’s disease. This angle is created by the intersection of the line connecting the proximal tibial metaphyseal border and the line perpendicular to the lateral border of the tibial bone [6]. A value greater than 16 degrees accurately predicts the progression of the proximal tibial varus deformity for which early treatment is recommended. The MMB is the angle formed by the intersection of a line parallel to the medial cortex of the proximal tibia with a line connecting the medial metaphyseal beak to the first line at the physis level. An MMB value greater than 122 degrees was reported to have an AuROC value of 0.983 for discriminating Blount’s disease from the physiologic bowlegs [7].

The diagnostic prediction model developed in this study includes both clinical and radiographic parameters to improve the model accuracy for diagnosing Blount’s disease. For practicality, the developed diagnostic model is presented with weighted scoring to improve the ease of use. Patients with a total score < 2.5 have a lower likelihood of Blount’s disease. Nevertheless, an annual follow-up is still recommended for physical examination. Patients with a total score from 2.5 to 5.5 were identified as the moderate-risk group, which is recommended for more frequent follow-up for physical examination and radiographic study. Patients with a total score > 5.5 should be strongly considered for a diagnosis of Blount’s disease, and an immediate referral to pediatric orthopaedists is recommended for treatment initiation.

This study has several strengths. First, the diagnostic prediction model developed in this study is the first diagnostic tool to use both the patient’s clinical and radiographic information, which shows excellent model discriminative performance. Second, this scoring system uses simple, well-defined parameters that are practical for primary care physicians who might not be able to efficiently interpret Blount’s disease radiographic changes [5]. There are also some limitations of our study that need to be disclosed and discussed. First, our model was developed from retrospectively collected data. Second, although the sample size in this study is relatively small, only minimal model optimism was detected from internal validation processes. Third, the proposed model wasn’t created using a cohort of the intended domain but rather using a case-control series. Consequently, our model cannot accurately predict the probability of Blount’s disease diagnosis, so positive and negative likelihood ratio (and their 95% confidence intervals) were presented instead. Finally, since internal validation of this model revealed minimal optimism, an external validation study should be conducted prior to clinical implementation.

## 5. Conclusions

The developed diagnostic prediction model for discriminating an early stage of Blount’s disease from physiologic bowlegs demonstrated high discriminative ability with minimal optimism. This model could assist primary care physicians in making an early diagnosis and treatment selection to improve the final outcome of Blount’s disease.

## Figures and Tables

**Table 1:** Demographic and Clinical Characteristics of the 79 Included Patients.

Patient Demographic	Mean	±SD
Age (month)	26.0	6.1
Gender (*n*, %)		
Male	48	60.8
Female	31	39.2
BMI ^1^ (kg/m^2^)	24.9	4.5
Laterality (*n*, %)		
Blount’s disease of right leg	9	11.4
Blount’s disease of left leg	19	24.1
Bilateral Blount’s disease	28	35.4
Bilateral physiologic bowlegs	23	29.1
FTA ^2^ (°)	11.6	5.7
MDA ^3^ (°)	12.4	3.6
MMB ^4^ (°)	122.9	6.1

^1^ BMI, Body Mass Index; ^2^ FTA, Femoro-Tibial Angle; ^3^ MDA, Metaphyseal-Diaphyseal Angle; ^4^ MMB, Medial Metaphyseal Beak angle.

**Table 2:** Demographic and clinical characteristics of the 158 lower extremities from 79 patients compared between those with Blount’s disease and those with physiologic bowlegs.

Characteristics (*n* = 158 Sides)	Missing Data		Blount Disease (*n* = 84 Sides)		Physiologic Bow-Leg (*n* = 74 Sides)		*p*-Value
	*n*	(%)	Mean	±SD	Mean	±SD	
Clinical characteristics							
Age (months)	0	0	27.0	5.2	24.9	6.9	0.030
Age ≥ 24 months (*n*, %)			57	67.9	37	50.0	0.024
Gender (*n*, %)							
Male	0	0	48	57.1	48	64.9	
Female	0	0	36	42.9	26	35.1	0.333
BMI ^1^	62	39.24	24.9	4.3	25.0	4.9	0.900
BMI ≥ 23 kg/m^2^ (*n*. %)			39	63.93	21	60.0	0.827
Laterality (*n*, %)							
Right	0	0	37	44.1	42	56.8	
Left	0	0	47	55.9	32	43.2	0.151
Radiographic Characteristics							
FTA ^2^ (°)	0	0	13.5	6.2	9.2	7.3	<0.001
FTA ≥ 5° (*n*, %)			75	89.3	49	66.2	<0.001
MDA ^3^ (°)	0	0	14.5	4.0	10.0	4.4	<0.001
MDA < 11° (*n*, %)			13	15.5	43	15.5	
MDA 11–16° (*n*, %)			40	47.6	27	36.5	
MDA > 16° (*n*, %)			31	36.9	4	5.4	<0.001
MMB ^4^ (°)	0	0	127.4	6.1	118.3	6.2	<0.001
MMB ≥ 122° (*n*, %)			64	76.2	18	24.3	<0.001

^1^ BMI, Body Mass Index; ^2^ FTA, Femoro-Tibial Angle; ^3^ MDA, Metaphyseal-Diaphyseal Angle; ^4^ MMB, Medial Metaphyseal Beak angle.

**Table 3:** Univariable and full model multivariable logistic regression analysis for an independent diagnostic predictor of Blount’s disease (imputed dataset *n* = 158).

Characteristics	Univariable Analysis				Multivariable Analysis			
(*n* = 158 sides)	uOR	95% CI		*p*-value	mOR	95% CI		*p*-value
Age ≥ 24 months	2.11	1.11	4.03	0.023	2.75	1.09	6.95	0.033
Male	0.72	0.38	1.37	0.322	0.70	0.27	1.79	0.459
BMI ^1^ ≥ 23 kg/m^2^	1.71	0.73	3.99	0.213	2.36	0.70	8.05	0.165
Right side	0.60	0.32	1.13	0.112	0.77	0.33	1.77	0.533
FTA ^2^ ≥ 5°	4.25	1.83	9.87	<0.001	1.37	0.45	4.19	0.580
MDA ^3^								
MDA < 11°	Ref.							
MDA 11–16°	4.90	2.23	10.79	<0.001	2.66	0.91	7.80	0.074
MDA > 16°	25.63	7.63	86.14	<0.001	11.65	2.44	55.63	0.002
MMB ^4^ ≥ 122°	9,96	4.79	20.68	<0.001	4.47	1.59	11.52	0.005

^1^ BMI, Body Mass Index; ^2^ FTA, Femoro-Tibial Angle; ^3^ MDA, Metaphyseal-Diaphyseal Angle; ^4^ MMB, Medial Metaphyseal Beak angle.

**Table 4:** Multivariable logistic regression analysis for an independent diagnostic predictor of Blount’s disease after backward elimination of preselected predictors with transformed coefficients and assigned scores (imputed dataset *n* = 158).

Characteristics	Multivariable Analysis				Score	
(*n* = 158 sides)	β	95% CI		*p*-value	Transformed β	Assigned score
Age ≥ 24 months)	1.05	0.15	1.94	0.022	1.34	1.5
BMI ^1^ ≥ 23 kg/m^2^	0.78	−0.30	1.87	0.154	1.00	1
MDA ^2^						
MDA < 11°	Reference					0
MDA 11–16°	1.16	0.17	2.16	0.022	1.49	1.5
MDA > 16°	2.60	1.10	4.11	0.001	3.34	3.5
MMB ^3^ ≥ 122°	1.50	0.58	2.43	0.001	1.93	2

^1^ BMI, Body Mass Index; ^2^ MDA, Metaphyseal-Diaphyseal Angle; ^3^ MMB, Metaphyseal Beak Angle.

**Table 5:** Distribution of Blount’s disease and physiologic bow-leg into low, moderate, and high-risk categories with model scoring, positive likelihood ratio (LR+), and negative likelihood ratio (LR−) with their 95% confidence intervals (CI).

Risk Categories	Score	Blount		Physiologic Bow-Leg		LR+	95% CI		LR−	95% CI		*p*-Value
		*n*	(%)	*n*	(%)							
Low risk	<2.5	6	7.1	31	41.9	0.17	0.06	0.45	5.86	2.27	18.01	<0.001
Moderate risk	2.5–5.5	38	45.2	41	55.4	0.82	0.46	1.45	1.22	0.69	2.18	0.462
High risk	>5.5	40	47.6	2	2.7	17.62	4.41	70.41	0.06	0.01	0.23	<0.001
Mean ± SE		5.2	0.2	2.5	0.2							<0.001

## Data Availability

The datasets used and/or analyzed during the current study are available from the corresponding author on reasonable request. The data are not publicly available due to their containing information that could compromise the privacy of research participants.
